# Spotted Wing Drosophila in Sweet Cherry Orchards in Relation to Forest Characteristics, Bycatch, and Resource Availability

**DOI:** 10.3390/insects9030118

**Published:** 2018-09-13

**Authors:** Ernest Ireneusz Hennig, Dominique Mazzi

**Affiliations:** Research Division Plant Protection, Agroscope, Müller-Thurgau-Strasse 29, 8820 Wädenswil, Switzerland; dominique.mazzi@agroscope.admin.ch

**Keywords:** forest, edge density, distance, *Drosophila suzukii*, bycatch, cherries

## Abstract

Forest vegetation is essential for the population development of the spotted wing drosophila (SWD). Yet, little is known of how the structure of surrounding forest areas influence the abundance of SWD within orchards. In this work, we use data from a field trial at five sites in Switzerland to analyse the relationship between the extent of forest area, its edge density, and its distance from the orchard with the occurrence of SWD in sweet cherry orchards in a Bayesian hierarchical model. Availability of cherries and bycatch were also included in the model to account for effects of resource availability and trap attractiveness, respectively. For all main effects and their interactions, we accounted for potential temporal changes by adding interactions with time. We found that the closer an orchard was to a forest, the more SWD were trapped within the orchard. However, the interaction of forest proximity with forest area caused a disproportionate decrease of SWD catches. Also, the within orchard variables, trap catches of other drosophilid flies and resource availability affected SWD trap catches, but their relation changed in the course of the experiment. The findings imply that reducing SWD occurrence in orchards and other crop fields requires not only the consideration of processes outside and within the host crop field, but also of temporally changing relationships between SWD and other factors.

## 1. Introduction

Landscape composition surrounding crops plays an eminent role for insect pest species and their antagonists. This role is generally advocated for antagonists (e.g., [1,2,3,4,5]), but it has been taken to a lesser extent into account for pests. In fact, landscape structures such as forests, shrub vegetation and flowering field margins provide shelter, connectivity, alternative habitats, and food sources for pests [6,7]. Consequently, the extent of these structures can foster pest species abundance and diversity [7,8,9,10,11]. In addition to the extent of landscape structures, their shapes can also influence pest colonization of host crops. Habitat shapes vary from linear to complex structures [12]. Complex structures can favour pest movement towards host crops by providing microhabitats that protect against unfavourable weather conditions and direct movement towards host crops [13].

Furthermore, the distance of landscape structures to host crops can influence their role for pests. Pest species can overcome large distances given habitat connectivity, weather conditions, alternative resources and enough time [8,11]. Pest species with limited dispersal ability, however, are more likely to occur in non-crop habitats close to host crop fields and launch periodic or regular invasions, while moving between non-crop habitats and crop fields [14]. The extent and shape of forest areas may thus be more relevant at closer distances for pest species with limited dispersal capabilities. Including information about the extent and shape of landscape structures on pest species, and the distance to host crops is therefore essential to boost the beneficial effects and at the same time mitigate the detrimental effects of any agro-environmental or biodiversity promoting schemes and related habitat manipulations intended to favour pest control [15,16]. Finally, biological control of pests and other pest management measures may not be sufficient to reduce significantly crop infestation at the landscape scale [17,18]. This is especially the case for seminatural crops such as high-stem fruit tree orchards, where many pest management measures are not applicable or not economically viable [19]. For example, the use of enclosure nets to protect high-stem fruit trees is not cost-effective relative to the product value. Increasing the effectiveness of biological control and pest management requires knowledge about the pest’s biology, which includes the role of landscape structures for dispersion and survival.

The spotted wing drosophila (SWD), *Drosophila suzukii* (Matsumura, 1931), is a polyphagous pest of stone and soft fruits in Europe, North and South America [20,21,22]. Since its first occurrence in Europe in 2008, SWD invaded almost all European countries. In Switzerland, it was first detected in 2011 [23]. Economic damage amounts to several hundred thousand Euros annually, while additional costs arise from pest management [24,25]. Current research on pest management focuses on chemical and mechanical control, trap efficiency, and the sensitivity of varieties to SWD infestation [26,27,28,29,30]. Recent findings highlighted the importance of wooded habitats [31,32,33,34]. To our knowledge, there is currently little information about whether distance from neighbouring non-crop habitats and extent of non-crop habitats affect the colonization of host crops by SWD [34,35,36,37], and no information about the effects of the shape of those non-crop habitats. Additionally, several trap types have been designed and tested with different attractants for their efficiency in catching SWD. Although different trap aspects have been shown to affect the efficiency of traps [26,38,39,40,41], a potential decay of the attractant and its role to attract SWD has not been considered. Attractants can undergo changes in their composition due to the effect of temperature and the biomass of captured insects accumulating over time, and hence alter the effectiveness of the trap.

This paper contributes to the current research on SWD by addressing relevant questions on the role of landscape metrics and local factors on the colonization of host crops by SWD. We assumed that the abundance of SWD increases with the area of forest and the complexity of forest shapes in the surroundings of suitable hosts, as well as with the abundance of suitable host fruits. We further predicted that this relationship depends on the distance of the forest areas from the host crop. We also expected fewer adult captures of SWD in traps with abundant captures of other insects.

## 2. Materials and Methods

### 2.1. Study Sites and Experimental Design

The study was carried out at five sweet cherry orchards between the 23rd of June (23rd calendar week) and the 3rd of August (31st calendar week) 2017, when between 3.8% (Wölflinswil) and 30.3% (Kaisten) of cherries showed colour-change. The study ended two weeks after the last harvest (29th calendar week). Two orchards were located in the canton (similar to county) of Zug (Notikon (N) and Rotkreuz (R)) and three in the canton of Aargau (Schupfart (S), Wölflinswil (W), and Kaisten (K)). Cherry trees in Notikon and Rotkreuz were high-stem orchards with trees standing 10–20 m (Notikon) and 5–12 m (Rotkreuz) apart. In Schupfart and Wölflinswil, trees were planted in spindle bush, and in Kaisten in Cordon formation. At all three orchards, rows were 4 m and trees 2.5 m apart. All orchards in the canton of Aargau except in Kaisten (12% canned cherries) produced exclusively dessert cherries. The two orchards in the canton of Zug produced cherries for processing. Orchards differed in terms of size and cherry trees density, with the largest orchard in Notikon being almost ten times larger than the smallest orchard in Rotkreuz (Table 1). Highest tree densities occurred in Schupfart and Kaisten, while Notikon had the lowest cherry tree density. Average temperature and relative humidity differed little between orchards during the sampling period. Lowest temperatures were measured at Schupfart (19.9 ± 0.3 ${}^{\circ }$C), and highest in Kaisten (20.4 ± 0.5 ${}^{\circ }$C). Temperatures at Wölflinswil (20.1 ± 0.4 ${}^{\circ }$C), Notikon (20.0 ± 0.4 ${}^{\circ }$C), and Rotkreuz (20.0 ± 0.2 ${}^{\circ }$C) were almost identical. Notikon (66.4 ± 5.2% RH) and Wölflinswil (71.9 ± 3.5% RH) were less humid than Kaisten (73.4 ± 2.8% RH), Rotkreuz (73.1 ± 2.1% RH), and Schupfart (73.2 ± 2.8% RH). Orchards differed further in terms of altitude with the highest orchard at Wölflinswil being located more than 100 m higher than the lowest orchard at Kaisten. Plant protection measures against SWD consisted of 2–3 applications of thiacloprid (Alanto, Bayer Schweiz AG) (Wölflinswil, Switzerland), acetamiprid (Gazelle, Stähler Suisse AG) (Kaisten, Schupfart, Rotkreuz, Notikon, Switzerland), spinosad (Audienz, Omya Schweiz AG) (Kaisten, Schupfart, Switzerland), and kaolin (Surround, Stähler Suisse AG) (Notikon, Rotkreuz, Switzerland). Orchards at Kaisten, Schupfart, and Wölflinswil were covered with a weather protection foil. At Schupfart, protective lateral nets with a mesh size of 0.8 mm were additionally installed during the sampling period. The nets likely accounted for the few captures. However, SWD were trapped within the orchard despite the nets, which is why we kept this orchard in the analysis. Kaisten and Wölflinswil had nets against birds, while Notikon and Rotkreuz had no physical protection measures.

**Figure A1:**
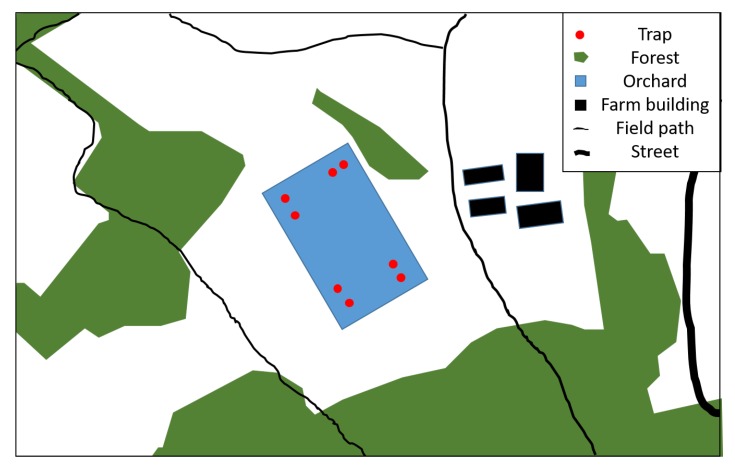
Drawing of the sweet cherry orchard, its surroundings and traps at Wölflinswil.

Two transparent traps closed with a red lid and baited with 80 mL of the commercially available liquid Gasser attractant (RIGA AG, Ellikon a.d. Thur, Switzerland) were mounted onto different cherry trees in each corner of the orchards at heights between 135–250 cm (for an example see Appendix A, Figure A1). Traps were emptied and replaced weekly. SWD, parasitic wasps, gnats (*Sylvicola* species), and other drosophilid flies were identified and counted. We included gnats in our study, because they were the most abundant species in the traps. Additionally, a LogTag HAXO-8 logger (MicroDAQ, Contoocook, NH, USA) was mounted next to traps in each corner, and temperature and humidity measurements were taken every 5 min throughout the sampling period. Cherries hanging on each tree provided with a trap were counted to evaluate local fruit availability.

### 2.2. Landscape Metrics

Geographical information about forests in the study regions was obtained from the Topographic Landscape Model (swissTLM3D 1.4 (2016)). Before analysis, forest shapes from recent satellite images surrounding field sites were visually compared with polygons from the swissTLM3D layer for equality using the web application from the Swiss Federal Geoportal [42]. Around each trap, buffer rings (i.e., circles), with radius ranging from 80–1000 m in 20 m intervals were constructed, and forest area and edge density for each interval calculated using the packages rgeos [43] and spatialEco [44] in R-Cran Version 3.4.4 [45]. For the calculation of edge density ED for buffer *j* at each radius we used the following equation:

(1) $${\mathrm{ED}}_{j}=\frac{\sum_{i=1}^{n}e_{ij}}{A_{j}}\cdot 10,000$$

with $e_{ij}$ = edge length (m) of forest patch *i* within buffer *j*, and $A_{j}$ = total area (m${}^{2}$) of buffer *j*. Spearman rank correlation coefficients between the number of SWD and results for landscape metrics were calculated at each interval. Landscape metrics with highest correlation coefficients were chosen for further analysis.

### 2.3. Mixed Model

A weighted linear Bayesian mixed model with negative binomial family, orchard as the only random (grouping) effect (*N* = 5), calendar week number (*t* = 9) as random slope, and *n* = 360 observations was applied. Differences in variance between orchards were accounted for by including SWD trap catches from each orchard as weights. Fixed effects considered the relationship of the sum of caught SWD for each trap on landscape metrics, reflecting the situation surrounding the orchards, and ecological variables, representing the situation at trap locations. The landscape metrics were (1) distance to the closest forest area; (2) percentage of forest area; and (3) edge density of forest patches. Ecological variables were (4) local fruit availability at trap location and (5) number of other caught insects excluding parasitic wasps. Other insects consisted almost entirely of other drosophilid flies and gnats from the genus *Sylvicola*.

All numerical and integer variables were standardized to μ = 0 and σ = 1 to aid in convergence of the algorithm and reduce correlation between group means and predictors. Collinearity was studied using Spearman correlation of posterior values from explanatory variables [46,47]. Because correlation analysis only considers bivariate relationships, we also included variance inflation factors (VIF). The presence of collinearity would increase the width of the highest posterior density intervals, which reflects uncertainty in parameter estimation [47]. The response variable, the number of caught SWD in 1 week, represents count data in a given time period. The occurrence of many zero and larger count values increases the variance and thus violates the condition for the Poisson model. These count data were modelled using a negative binomial distribution that has an additional shape parameter that accounts for the variance [48].

We used the brm function in the R-package brms version 2.3.0 [49] in R-Cran [45]. The brms package is a front-end for the software Stan, which has the Hamilton Monte Carlo algorithm implemented. This algorithm explores the probability space of model parameters more thoroughly than the Gibbs sampler [50,51]. Bayesian mixed models models provide less biased estimates when sample size is small than their corresponding frequentist approaches [52,53,54]. The success of parameter space exploration consisted of inspecting trace and density plots. The potential scale reduction factor ($\hat{R}$) represents the ratio of average sample variance in each chain to the pooled sample variance from all chains. Deviations from 1 indicate that the chains do not reach a common distribution and the model needs to be adjusted [55]. The effective sample size ($n_{eff}$) gives the number of independent draws from the chain to estimate parameter values. Values larger than 2000 are considered sufficient for inference [47,56]. Autocorrelation plots provide futher support for the reliability of independent draws. Priors were constructed after data collection but before analysis and based on previous publications and expert opinion. Priors and diagnostic plots are presented in the Appendix. We further present posterior means of parameters and high-density posterior intervals of fixed effects coefficients from a model with 10,000 iterations, 3 chains, and a warmup of 2000 iterations. We discarded each 5th value to reduce autocorrelation between posterior values. All parameters are initialized to zero.

## 3. Results

### 3.1. Trap Content

**Figure 1:**
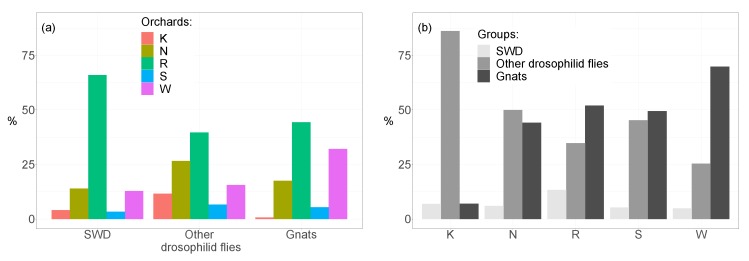
SWD, other drosophilid flies, and gnats between (**a**) and within (**b**) orchards. Parasitic wasps are not shown due to their low number. Abbreviations for orchards: K = Kaisten, N = Notikon, R = Rotkreuz, S = Schupfart, W = Wölflinswil.

In total, we caught 158,823 insects during 9 weeks. Considering catches from all orchards together, gnats were the most common insects recovered in the deployed traps (52.1%), followed by drosophilid flies (excluding SWD, 38.6%), and SWD (9.2%). Parasitic wasps that attack drosophilid flies made up only 0.04% of the total and consisted of species belonging to 5 families (Braconidae, Diapriidae, Figitidae, Proctotrupidae, and Pteromalidae). Among orchards, the distribution of SWD, other drosophilid flies, and gnats was similar with most catches in the orchard at Rotkreuz and least at Schupfart (Figure 1). In contrast, the contribution to the total catches differed considerably for the three taxa among the five orchards. Catches at Kaisten were dominated by drosophilid flies other than SWD (86.2%, Figure 1). SWD and gnats each made up only about 6.5% of the catches. At Notikon, other drosophilid flies than SWD (49.9%) and gnats (44.1%) were caught with similar frequency, while SWD (6.0%) was similarly seldom as in Kaisten. For the orchards at Rotkreuz and Wölflinswil, the distribution among the three groups followed the same pattern. Gnats were the most common insects in traps, followed by other drosophilid flies, and SWD.

### 3.2. Time Series

**Figure 2:**
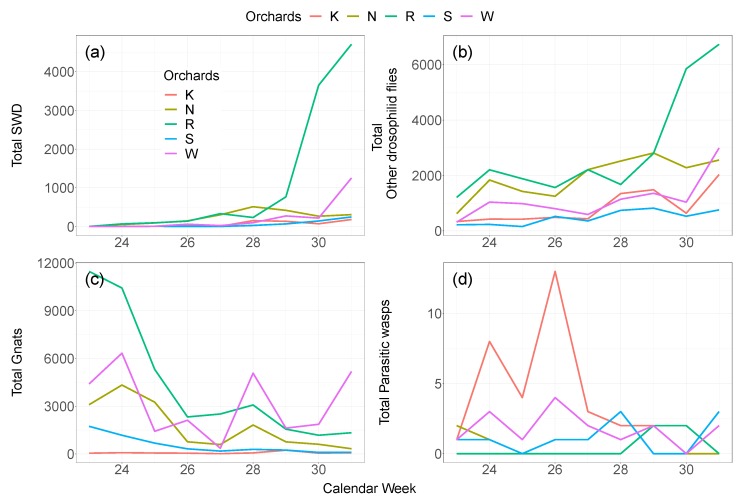
Number of SWD (**a**); other drosophilid flies (**b**); gnats (**c**); and parasitic wasps (**d**) from 23rd to 31st calendar week at the five orchards. Note the different dimensions of the *y*-axes.

The number of caught SWD and other drosophilid flies increased in the course of the experiment, especially in the orchard at Rotkreuz (Figure 2). Gnats and parasitic wasps showed the opposite pattern, with most individuals in traps at the beginning of the experiment. Again, Kaisten showed little variation in time for the occurrence of gnats, but had the highest captures of parasitic wasps throughout the first weeks (24th–26th). During the experiment, almost no parasitic wasps were found at Rotkreuz, which stands in contrast to the largest abundance of SWD, other drosophilid flies and gnats in this orchard.

### 3.3. Landscape Metrics

**Figure 3:**
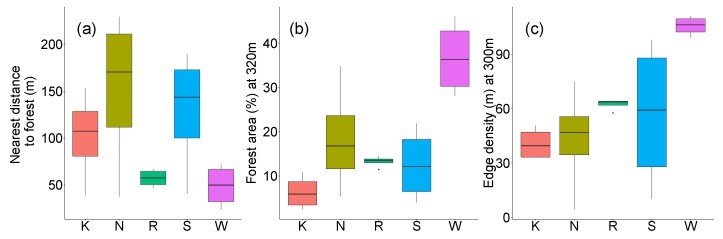
Distance to forest (**a**), forest area (**b**), and edge density (**c**) for the five orchards. Note the different ranges of the *y*-axes.

The three landscape metrics distance to forest (m), forest area (%), and edge density (m) varied among orchards. Distance to forest was smallest for all traps at Wölflinswil and Rotkreuz (49 m and 57 m, respectively). In contrast, distance to forest showed larger variation among traps at Kaisten, Notikon, and Schupfart, but was in general longest at Notikon (>150 m) (Figure 3a). The smallest extent of forest area around traps was found at Kaisten (6.3%), which was six times less than Wölflinswil, the field site with the largest forest area (Figure 3b). Notikon, Rotkreuz, and Schupfart had on average more than 12% of forest area around traps, but below 20%. The situation for edge density was similar for the lowest (Kaisten, 40.7 m) and highest values (Wölflinswil, 105.5 m), while Rotkreuz had larger edge density of forest patches (62.4 m) than Schupfart (56.6 m) and Notikon (43.3 m) (Figure 3c).

**Figure 4:**
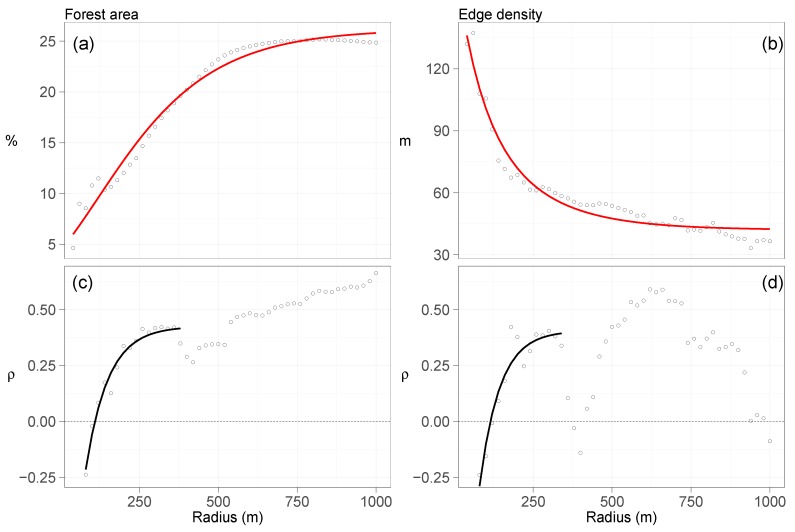
Forest area, edge density, and their Spearman correlation coefficients (ρ) with SWD at distances from 80–1000 m in 20 m intervals. Fitted red lines in (**a**,**b**) represent results from Gompertz functions (Equation (2)). The black lines in (**c**,**d**) are results from limited growth models (Equation (3)).

The percentage of forest area pooled from all field sites increased with the buffer radius until 500 m, when the curve started to level off at 25% forest area (Figure 4a). Edge density declined with increasing radius, starting at an edge density above 120 m at the lowest radius (80 m) and ending below an edge density of 40 m at 1000 m radius (Figure 4b). While the relationship between forest area and buffer radius was smooth, there was more variation in the relationship between edge density and buffer radius. Both curves can be described using a Gompertz function:

(2) $$\begin{array}{ccc} f_{PA}\left(x\right) & = & 262e^{-2.79e^{-x}}\\ f_{ED}\left(x\right) & = & 42.01e^{1.43e^{-x}} \end{array}$$

Spearman correlation coefficients were similar between percentage (range [−0.24;0.66]) and edge density of forest areas (range [−0.24;0.59]). Lowest correlations were at the smallest radius (80 m), while largest correlations at 1000 m radius for percentage of forest areas (Figure 4c), and at 620 m radius for edge density (Figure 4d). Correlation coefficients increase exponentially until 360 m in percentage and 320–340 m in edge density (Figure 4c,d). Edge density, however, has a bimodal distribution. The first peak is at 300 m and covers the range between 80–380 m, and the second peak at 620 m with the range of 400–1000 m. The exponential relationship between radius and correlation coefficients for the first part of each landscape metric describe models for limited growth of the form:

(3) $$\begin{array}{ccc} f_{PA}\left(x\right) & = & 0.426-1.552\cdot e^{-e^{-4.262}\cdot x}\\ f_{ED}\left(x\right) & = & 0.406-2.025\cdot e^{-e^{-4.157}\cdot x} \end{array}$$

with *x* = radius, PA = percentage, and ED = edge density of forest areas. Values from radius for each landscape metrics were chosen from the corresponding highest correlation coefficients within the range of the fitted line. For forest area, this was 320 m, while for edge density 300 m. We did not consider correlation coefficients beyond the fitted line from the non-linear regression model.

### 3.4. Covariables

**Figure 5:**
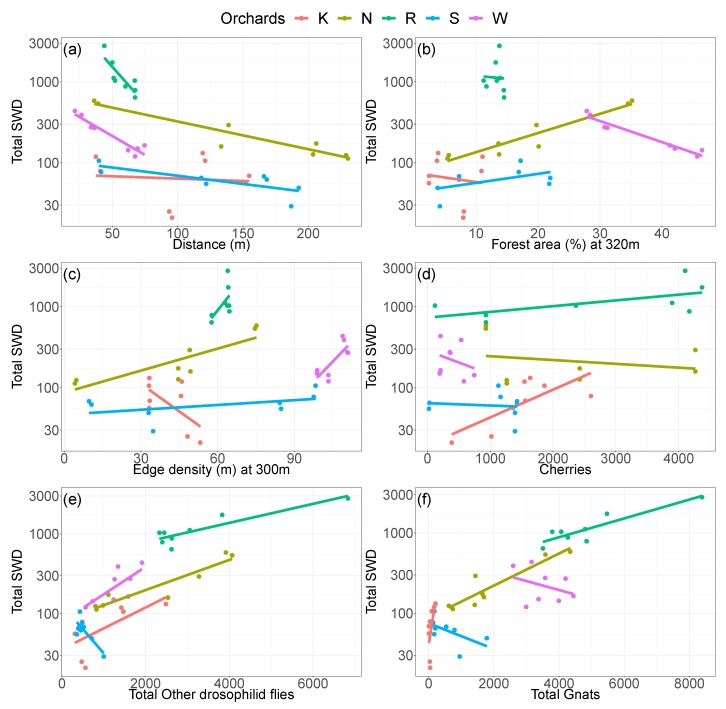
Scatterplots between trap catches of SWD and covariables at each orchard. SWD catches are log${}_{10}$-transformed. Lines are taken from ordinary regression analyses for each orchard.

Considering independent regression models for each field site, effects of covariables differed among field sites (Figure 5a–f). Although the total number of SWD declined with distance to forest areas at all orchards, the relationship was strongest at Rotkreuz and least pronounced at Kaisten (Figure 5a). Similarly, positive relationships were found for almost all orchards between the total number of caught SWD and bycatch (i.e., other drosophilid flies and gnats) (Figure 5e,f). At Schupfart and Wölflinswil, however, the total SWD number decreased with the total number of other drosophilid flies and gnats, respectively. Forest area, edge density, and the number of cherries showed a larger variation than distance to forest and bycatch in terms of the directions of the relationships (Figure 5b–d). Pooling observations from all orchards together, only other drosophilid flies, gnats, and the distance to forest showed obvious relationships with the total number of caught SWD (line not shown). The number of caught SWD declined with increasing distance to forest, but increased with other drosophilid flies and gnats.

### 3.5. Model Results

**Figure A2:**
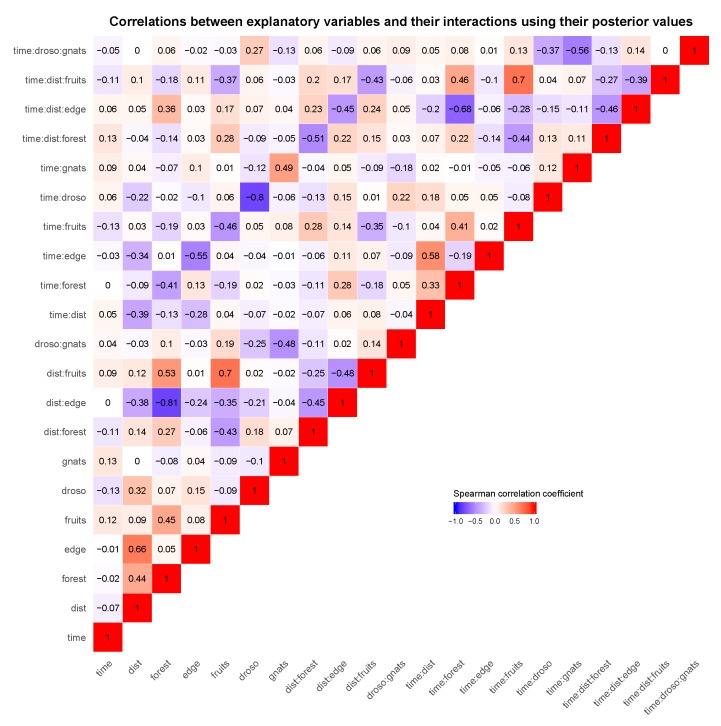
Spearman correlation coefficients between explanatory variables using posterior values.

**Figure A3:**
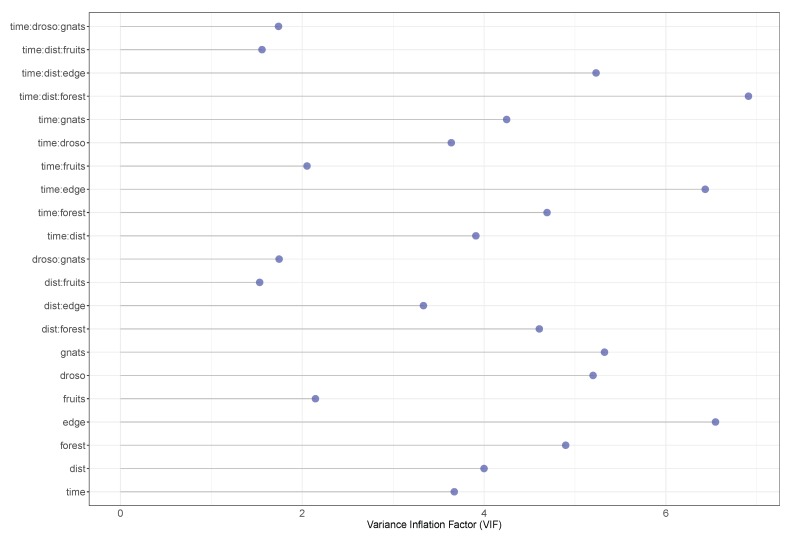
Variance inflation factors for explanatory variables. All values are below VIF = 10.

**Figure A4:**
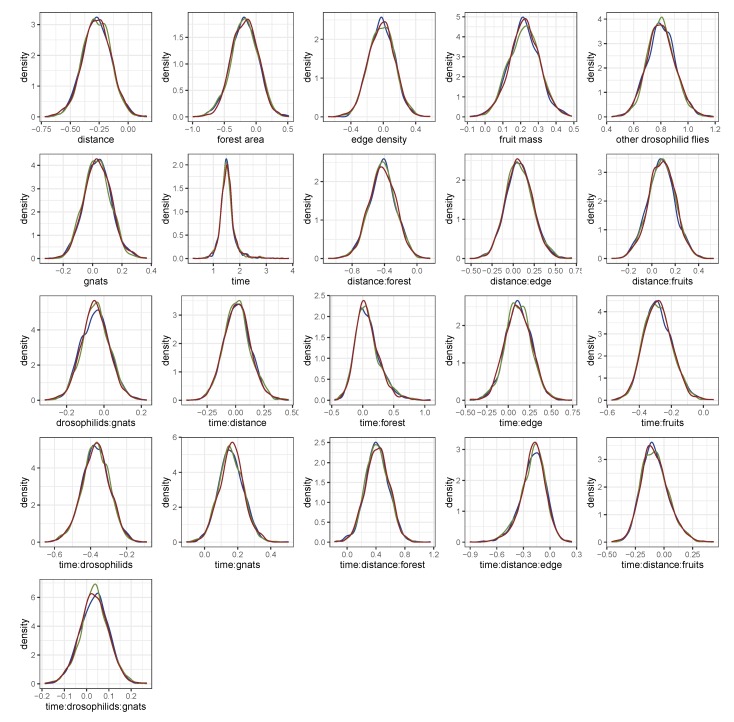
Density plots of posterior draws from each chain for parameters of the model variables. Please note that the variable time is represented by calendar weeks and burnins are excluded.

**Figure A5:**
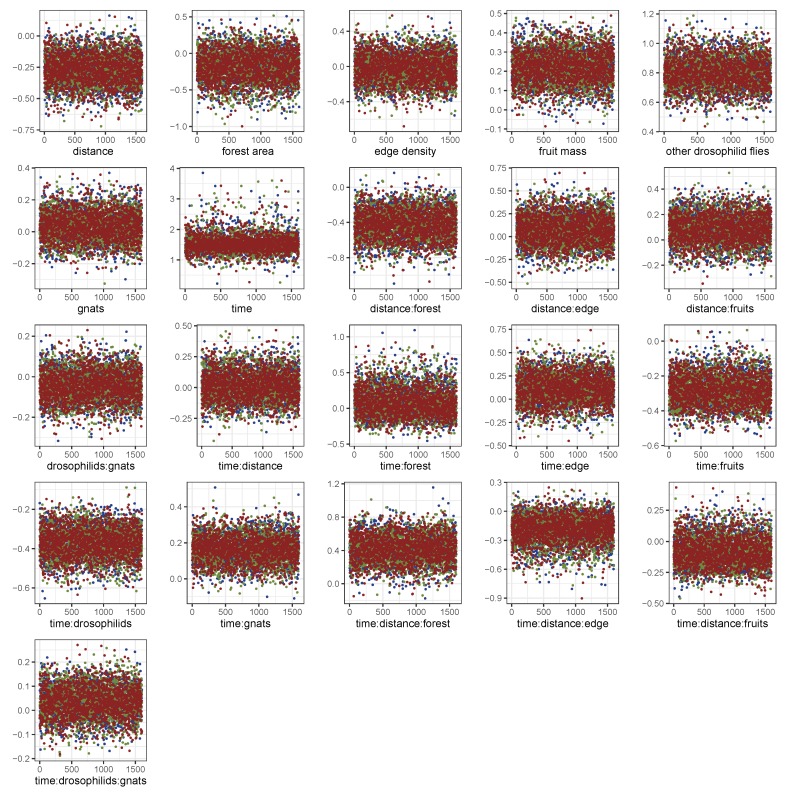
Traceplot showing parameter values (points) chosen by the three chains (colors). Please note that the variable time is represented by calendar weeks and burnins are excluded.

**Figure A6:**
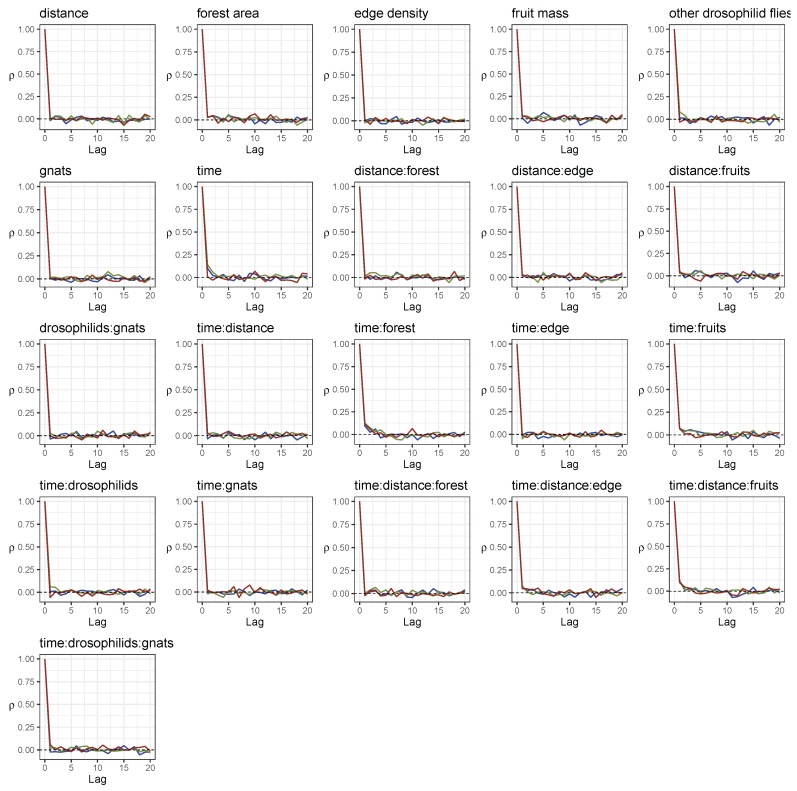
Autocorrelation plots from posterior draws of parameters in each chain. Please note that the variable time is represented by calendar weeks and burnins are excluded.

Spearman correlations between explanatory variables using their posterior values revealed 4 large correlations (ρ≥|0.7|) out of 210 correlations (Appendix C, Figure A2). The variance inflation factors were all below the critical value 10 (Appendix C, Figure A3), implying little effects on the width of the highest posterior density intervals (HDPI). The parameter traceplots indicate that the 3 chains explored the parameter space thoroughly (Appendix D, Figure A4, Figure A5 and Figure A6). Density plots of coefficients show approximately normal distributions, indicating limitation of coefficients to a small range of likely values. The smallest effective sample size was 3318 and the scale reduction factor $\hat{R}$ = 1.0 for all random and fixed effects (Table 2).

The highest posterior density intervals (HPDI) of the lower 2.5% and upper 97.5% for distance to the closest forest were negative, but did include zero, suggesting that the number of caught SWD did not change with the distance to the closest forest. The interaction between distance to the closest forest and the percentage of forest area, however, was negative. This implies that with increasing distance to the closest forest, the effect of percentage of forest area on SWD trap catches declines. The positive lower and upper 95% HPDI for other drosophilid flies were positive, indicating an increase of caught SWD with the number of other drosophilid flies. The number of cherries, trap catches of gnats, percentage of forest area, edge density, and the other interaction terms had no compelling effects on the abundance of SWD in traps.

Considering the interaction of main effects with time, the confidence intervals for posterior means of trap catches of other drosophilid flies and gnats, and the interaction between distance to forest and forest area did not include zero and hence showed an effect on SWD. Spotted wing drosophila thus declined with increasing number of cherries and other drosophilid flies in the course of the experiment, while it increased with gnats. Also, the interaction between distance to forest and forest area caused a disproportionate increase of SWD over time.

## 4. Discussion

We used a holistic approach for understanding SWD colonization of sweet cherry orchards, analyzing forest distance and metrics at the landscape scale, and bycatch and resource availability at the local scale. Our results indicate that decreased forest proximity is associated with increased SWD trap captures, but the interaction of forest proximity with forest area causes a disproportionate decline of SWD catches. Also, an increasing number of other drosophilid flies as well as local resource availability was positively correlated with the number of trapped SWD. However, in the course of the experiment the relationships changed, which indicates their temporal dependence.

Few earlier studies reported an association of SWD with woody habitats [32,34]. The infestation of orchards requires, therefore, the forest to be temporarily less attractive than the orchard and the orchard to be in reaching distance of the forest. In other dipteran species, single individuals can overcome distances of more than 1 km [57,58,59], but most likely cover smaller distances [60,61]. The decline of SWD in orchards with increasing distance to forests in our analysis underlines the importance of the proximity of forests or similar woody vegetation structures [62] and adds to recent evidence that SWD has a relatively small dispersal range [61]; [63]. The availability of alternative resources and the scope for buffering adverse weather periods correlate with the size of forests (and alternative structures) [64]. Larger areas of vegetation have longer edge zones that provide more likely abundant microhabitats for alternative host plants as well as refuges [14,65,66,67,68]. Large vegetation structures thus foster the growth of SWD populations prior to the ripening of cultivated host fruits [69]. Consequently, abundant catches occur in the orchard regions facing forest areas. The lack of a similar effect at distant forest areas supports a limited dispersion ability of SWD. The large number of SWD in orchards with closer forest areas, however, declines with time. This decline suggests a break in the continuous SWD movement from forests to orchards, and may be related to the increased attractiveness of other, later maturing host crops nearby, or of the forest habitat. However, we have not estimated population size of SWD in nearby host crops or forests to corroborate the assumption.

Forest edges are ecotones located between a forest and another habitat [68]. With characteristics of both habitats, they are more complex than the forest and the neighbouring area. This large complexity correlates often with a higher diversity of plants and insects [64,67,68,70]. Moreover, larger edge complexity provides tunnel-like structures that facilitate dispersal of insects [13]. The lack of evidence for edge effects on the occurrence of SWD in the studied orchards suggests that this pest species is little affected by edge complexity. Despite the important role of edges in landscapes on the abundance and movement of insects [13,68], many studies find little support for edge-mediated effects [71]. Vegetation composition and structure of forest edges vary, resulting in hard and soft edges [72,73]. Soft edges have smoother abiotic gradients than hard edges, which are more exposed to insolation and winds [74]. SWD populations thrive at mild temperatures, high humidity, and in wind protected locations [75,76]. Consequently, soft edges have the potential to favour, while hard edges limit the development of SWD populations. The presence of both types of edges can explain the absence of an edge effect on the abundance of SWD in orchards in our study.

Larger quantities of fruits attract more flies due to the maximization of population development [77]. Our study provides contradictive results, because fewer SWD were caught at places with high cherry fruit availability at the end of the experiment. The reason for the temporal change in the attractiveness of cherry fruit quantity may be related to the relative attractiveness of the trap and the local abundance of SWD. After colonization of the orchard and the increase of the population, the local availability of many cherries reduces the relative attractiveness of the trap attractant and weakens competition for oviposition sites among conspecifics [78]. On the other hand, the availability of few cherries can foster competition between individuals for oviposition sites and thus make the trap more attractive to SWD when population size in the orchard is increasing. As a result, more SWD can be expected in traps in orchard regions with fewer cherries when population size is large than in regions with many cherries. Similar observations have been made with apple maggot flies, where competition between the natural apple odours and a synthetic blend has been found [79].

Among the several drosophilid flies occurring in Central Europe, SWD is the only species reproducing in ripening and ripe cherries and other stone fruits to such an extent that it causes large economic damage [24,25,80,81]. Cherries and other fruit hosts provide, however, resources to other drosophilid species as well. Despite overlapping breeding sites, the ripeness stage of cherries reduces the overlap of ecological niches of drosophilid flies and allows the presence of several drosophilid species in the course of ripening [82]. The susceptibility of fruits to SWD increases with ripeness stage [83]. In turn, the ripeness of fruits is related to the emission of volatiles, especially ethanol [84]. Ethanol emission can be escalated by fermentation processes due to the activity of bacteria and yeasts [85], which serve also as food for adult SWD and other drosophilid species [86,87]. Spotted wing drosophila responds positively to high concentrations of ethanol, which also attracts other drosophilid species that use cherries in advanced senescence stages for reproduction [88,89]. The competition between SWD and other drosophilid species can increase with the abundance of the latter and imply the displacement of SWD. Although interspecific competition has been shown elsewhere [90,91,92], local coexistence of different drosophilid species is often facilitated by spatial and temporal resource partitioning. In particular, the sclerotized ovipositor of SWD allows the exploitation of host fruits in early ripeness stages. Successive infestation of bacteria and yeast does not only accelerate the fermentation process, but also facilitates access to the host fruit for other drosophilid flies which are not capable to penetrate the skin of host fruits in early ripeness stages. This makes the displacement of SWD by other drosophilid species unlikely [93,94,95,96,97,98].

The accumulation of bycatch can modify the volatile composition such that the attractiveness of traps is reduced. Gnats of the genus *Sylvicola* accounted for the largest fraction of bycatch in traps during the experiment, hence we assumed that the large contribution to the trap content can alter the attractiveness of the trap. Because the number of gnats increased with SWD as the experiment progressed, there was no detrimental effect of gnats on the attractiveness of traps to SWD. We assume that this positive relationship is determined by the importance of forest areas for both, gnats and SWD, and the increase of SWD population during the experiment. In fact, the larvae of the genus *Sylvicola* develop preferentially in decaying, fermenting plant material, which is correlated with forest area [99,100]. Indeed, the majority of SWD and gnat catches were found at the orchards with the largest extent of forest area, which this positive relationship. This positive relationship appeared, however, not until the SWD population reached a large size, which reflects the dynamic nature of relationships between SWD and co-occurring taxa.

## 5. Conclusions

The landscape matrix plays an important role in shaping the occurrence of SWD in orchards. The distance of orchards to the closest forest patch as well as the extent of forest area in the surroundings of orchards determine the abundance of SWD and thus the severity of infestation. Effective control of SWD requires the consideration of these landscape metrics. However, the lack of an effect of edge density suggests that detailed studies at habitat scale are required to understand how characteristics of forests and other vegetation structures influence orchard colonization. In addition, a complex picture of interactions between SWD, co-occurring taxa, host fruit availability, and landscape metrics with time may explain the varying colonization patterns and success to control SWD, while also emphasizing the need to consider multiple sites in the study of pest-landscape relationships.

## Figures and Tables

**Table 1:** Field site characteristics. Exposition: E = East, S =  South, SSW = South Southwest. min/max = min and max distance between trap pairs.

Field Site	WGS84 (Lat/Lon)	m (a.s.l.)	Size (m${}^{2}$)	Cherry Trees	Varieties	Exposition	min/max (m)
Kaisten (K)	47.53572/8.03969	380–400	10,500	1005	9	E	33.9/189.1
Notikon (N)	47.21712/8.53908	510–550	31,200	230	33	S	87.2/206.9
Rotkreuz (R)	47.13200/8.42231	500	3000	64	2	-	8.4/90.3
Schupfart (S)	47.51972/7.95958	470–480	19,000	2080	8	SSW	132.0/250.8
Wölflinswil (W)	47.45926/7.98005	580–590	9500	800	8	E	51.9/132.3

**Table 2:** Posterior means and their lower (95%l) and upper (95%u) high density posterior intervals from a negative binomial Bayesian mixed model with *N* = 5 groups and *n* = 360 observations. Variables not including zero in the intervals are highlighted in bold. Abbreviations: dist = distance to nearest forest, forest = forest area, edge = edge density of forest patches, fruits = availability of cherries, droso = abundance of other drosophilid flies, gnats = abundance of gnats from the genus *Sylvicola*, time = calendar week, $\mu_{0}$ = mean intercept from orchards, $\mu_{1}$ = mean slope from time, ρ = correlation between intercept and slope, $n_{eff}$ = number of independent a posteriori values drawn from chains, $\hat{R}$ = scale reduction factor.

	Mean	95%l	95%u	$n_{\mathrm{eff}}$	$\hat{R}$
**Population main effects**					
**dist**	**−0.27**	**−0.51**	**−0.03**	**4800**	**1.000**
forest	−0.18	−0.61	0.21	4135	1.000
edge	−0.02	−0.33	0.29	4800	1.000
**fruits**	**0.22**	**0.05**	**0.39**	**4567**	**1.000**
**droso**	**0.80**	**0.60**	**1.01**	**4499**	**1.000**
gnats	0.03	−0.14	0.22	4682	1.000
**time**	**1.53**	**1.04**	**2.27**	**3866**	**1.000**
**dist:forest**	**−0.43**	**−0.76**	**−0.10**	**4425**	**1.000**
dist:edge	0.07	−0.23	0.40	4496	1.000
dist:fruits	0.08	−0.15	0.31	4051	1.001
droso:gnats	−0.05	−0.19	0.10	4800	1.000
**Population main effects in time**					
time:dist	0.01	−0.21	0.25	4640	0.999
time:forest	0.08	−0.25	0.54	3409	1.001
time:edge	0.12	−0.19	0.41	4800	1.000
**time:fruits**	**−0.28**	**−0.44**	**−0.11**	**3648**	**1.000**
**time:droso**	**−0.37**	**−0.53**	**−0.23**	**4572**	**1.000**
**time:gnats**	**0.16**	**0.02**	**0.31**	**4800**	**1.001**
**time:dist:forest**	**0.42**	**0.10**	**0.73**	**4800**	**1.000**
time:dist:edge	−0.19	−0.51	0.05	3900	1.000
time:dist:fruits	−0.08	−0.29	0.18	3580	1.000
time:droso:gnats	0.03	−0.09	0.16	4622	1.001
**Random effects**					
$\mu_{0}$	1.35	0.55	3.37	4397	1.001
$\mu_{1}$	0.50	0.04	1.92	3318	1.001
ρ	−0.13	−0.84	0.66	4729	1.000

## Abbreviations

The following abbreviations are used in this manuscript:

SWD	spotted wing drosophila
PA	percentage of forest areas
ED	edge density of forest patches
RH	relative humidity
swissTLM3D	Swiss Topographic Landscape Model

## Appendix B. Priors

**Table A1:** Priors of the Bayesian mixed model for parameters and their explanations. For all other parameters, the default priors of the function brm in the R package brms version 2.3.0 were used. Please note that all explanatory variables were standardized to mean μ = 0 and standard deviation σ = 1 before the analysis. $N(\mu ,\sigma^{2})$ = normal distribution with mean μ and variance $\sigma^{2}$, C(a,b) = Half-cauchy distribution with location *a* and scale *b*, G(k,θ) = Gamma distribution with shape parameter *k* and scale parameter θ, LJK(ν) = Lendowski-Kurowicka-Joe prior with parameter ν for correlation matrices.

Variable	Prior	Description
intercept	N(5,2)	The response variable is not standardized, hence the intercept cannot be 0. The prior mean was chosen from the mixed model R>lme4::glmner.nb(SWD∼1+(1+time|orchard)) using the package lme4 in R-Cran
dist	N(−1,0.5)	Previous studies found decreasing number of caught spotted wing drosophila with increasing forest distance from host crops [34,35,36].
forest area	N(0.5,1)	Larger extent of forest area provides more resources and refuge than smaller area [37], but sweet cherries can still be more attractive.
edge	N(1,0.75)	Higher values of edge density reflect more irregular shapes of forest patches. Such irregular shapes provide microhabitats that are less exposed to direct sunlight and wind than regularly shaped patches (e.g., rectangles, circles) [13]. We expect therefore more spotted wing drosophila flies (SWD) with higher values of edge density.
fruits	N(0.5,0.5)	Resource availability is expected to influence the number of SWD. More cherries should attract more individuals, however, plant protection measures can distort the relationship. Additionally, we counted cherries from trees with traps, but did not consider adjacent trees. We decided to use a weak prior for the parameter that assumes a positive relationship.
other drosophilid flies	N(0.75,0.5)	We assumed a positive relationship between SWD and the number of other drosophilid flies, because the drosophilid flies caught in the trap together with SWD habe the potential to share the same resource. We assigned this parameter a normal distribution with a positive mean, but also larger standard deviation due to a potentially deteriorating effect of increasing biomass in the trap on the attractant.
gnats	N(0.75,0.5)	See other drosophilid flies.
time	N(3,1)	Monitoring data in Switzerland (unpublished) showed an increase of SWD catches until autumn. The experiment started in early summer and ended in the summer. We expected therefore an increase of SWD throughout the experiment.
$\mu_{0}$ (mean intercept)	C(0,5)	The variance can only be positive, which is why we used a weakly, half-cauchy prior for the standard deviation of the mean intercept [101,102].
$\mu_{1}$ (mean slope)	C(0,5)	see $\mu_{0}$
ρ (correlation)	LKJ(2)	The LKJ prior for the correlation matrix. We reflected the belief that there is little correlation between intercepts and slopes, choosing the value ν = 2 ([103], p. 189).
α (shape parameter)	G(0.01,0.01)	We assigned a wide gamma prior for the shape parameter of the negative binomial distribution, which is the default procedure ([49], p. 4).

## Appendix D. Monte Carlo Markov Chain Diagnostics

### Appendix D.1. Density Plot

The density plots of the posterior draws of parameters show no multiple modes, which implies that we can trust estimates and highest posterior density intervals. Multiple modes would indicate that other parameter values are also likely.

### Appendix D.2. Trace Plot

The trace plot shows the values of the parameter sampled during the runtime of each chain. The chains cover a larger range of parameter values as expected. Results from the Bayesian mixed model using the negative binomial family. All chains are intermixing and covering a larger area of parameter space.

### Appendix D.3. Autocorrelation Plot

The autocorrelation plots show correlation coefficients of parameter posterior draws at each lag for each chain. The lines drop quickly to zero in all plots, indicating that there is almost no correlation among parameter posterior draws in each chain.
